# Controversy surrounding the increased expression of TGFβ1 in asthma

**DOI:** 10.1186/1465-9921-8-66

**Published:** 2007-09-24

**Authors:** Ynuk Bossé, Marek Rola-Pleszczynski

**Affiliations:** 1 Immunology Division, Department of Pediatrics, Faculty of Medicine, Université de Sherbrooke, Sherbrooke, QC, Canada

## Abstract

Asthma is a waxing and waning disease that leads to structural changes in the airways, such as subepithelial fibrosis, increased mass of airway smooth muscle and epithelial metaplasia. Such a remodeling of the airways futher amplifies asthma symptoms, but its etiology is unknown. Transforming growth factor β1 is a pleiotropic cytokine involved in many fibrotic, oncologic and immunologic diseases and is believed to play an essential role in airway remodeling that occurs in asthmatic patients. Since it is secreted in an inactive form, the overall activity of this cytokine is not exclusively determined by its level of expression, but also by extensive and complex post-translational mechanisms, which are all importanin modulating the magnitude of the TGFβ1 response. Even if TGFβ1 upregulation in asthma is considered as a dogma by certain investigators in the field, the overall picture of the published litterature is not that clear and the cellular origin of this cytokine in the airways of asthmatics is still a contemporaneous debate. On the other hand, it is becoming clear that TGFβ1 signaling is increased in the lungs of asthmatics, which testifies the increased activity of this cytokine in asthma pathogenesis. The current work is an impartial and exhaustive compilation of the reported papers regarding the expression of TGFβ1 in human asthmatics. For the sake of comparison, several studies performed in animal models of the disease are also included. Inconsistencies observed in human studies are discussed and conclusions as well as trends from the current state of the litterature on the matter are proposed. Finally, the different points of regulation that can affect the amplitude of the TGFβ1 response are briefly revised and the possibility that TGFβ1 is disregulated at another level in asthma, rather than simply in its expression, is highlighted.

## Transforming growth factor β1

Transforming growth factor (TGF)β1 is an intercellular signaling molecule that demonstrates a plethora of biologic effects in both *in vitro *and *in vivo *contexts. It was first isolated and characterized in platelets in 1983 [1] and is now the prototype member of a superfamily of cytokines, which actually counts 33 members in man [2]. For taxonomic purpose, members of the TGFβ superfamily are further divided into subgroups, and together with its two closest homologues TGFβ2 and TGFβ3, TGFβ1 forms the TGFβ subfamily.

TGFβ1 is synthesized as a prepropeptide of 390 aa and is encoded by a 7-exon gene (*TGFB1*) localized on chromosome 19q13.2. Several genetic studies have associated some of the common single nucleotide polymorphisms (SNP) found in the *TGFB1 *gene or its promoter with asthma phenotypes, supporting its potential role in the pathogenesis of this disease [3-8]. The gene is ubiquitously expressed, but its level of expression is transcriptionally and post-transcriptionally regulated. The maturation, expression and activation of the protein are also subject to extensive and complex post-translational regulation. Following its cleavage from the 29 aa signal peptide, the propeptide homodimerizes and both protomeres are cleaved by furin, a ubiquitous subtilisin-like proprotein convertase localized in the *trans*-Golgi network, at a canonic RX(K/R)R furin cleavage site found at position 275–278 on the TGFβ1 propeptide [9]. This proteolytic maturation generates the homodimeric mature protein, in which each of the 112-aa long protomeres remains associated due to hydrophobic interactions and a disulfide bridge [10]. This active form of TGFβ1 demonstrates a short half-life (normally less than 3 min) in cell-free systems [11]. To overcome its lability in *in vivo *conditions and to avoid premature binding with its cognate cell-surface receptor, TGFβ1 remains non-covalently associated with its propeptide latency-associated peptide (LAP). This post-translational modification renders TGFβ1 inactive during and after the secretory process. Latent TGFβ1 can also be secreted as a larger 180–210-KD multi-protein complex, which includes, in addition to the 75-KD LAP and the 25-KD mature TGFβ1 protein, the glycosylated 125–190-KD latency TGFβ binding protein (LTBP) [12].

Extracellular activation of latent TGFβ1 occurs through different mechanisms including: 1- proteolytic dissociation from LAP by the urokinase plasminogen activator (uPA)/plasmin system [13,14], or by other proteases such as metalloproteinase (MMP)-2 [9], MMP-9 [15,16] and the lysosomal serine protease cathepsin D [13]; 2- conformational alteration in its structure by thrombospondin [17] or integrins such as the αvβ6 integrin [18,19]; 3- oxidation and nitrosylation [20,21]; 4- removal of carbohydrate structure on LAP by glycosidases such as sialidase [22]; 5- integrin αvβ8-mediated latent TGFβ1 recruitment to the cell membrane for membrane type 1 (MT1)-MMP-dependent proteolytic activation [23]; and 6- extremes of pH or high concentrations of urea [24]. Mannose 6-phosphate/insulin-like growth factor II (IGF-II) receptor [25,26] as well as integrins α8β1 [27] and αvβ1 [28] also bind latent forms of TGFβ1 and are thus believed to target the latent complex on the surface of cells for subsequent proteolytic activation and ensuing binding to its signaling receptor. TGFβ1 is also a heparin binding growth factor (HBGF) [29,30]. Consequently, its binding availability for cell surface receptors is regulated extracellularly by heparan sulfate proteoglycans (HSPG). Whereas certain proteoglycans, such as betaglycan and endoglin [31], facilitate TGFβ1 binding to its receptors; others, such as biglycan, fibromodulin and decorin, sequester TGFβ1 in the ECM [32,33]. In addition, certain enzymes that cannot activate latent TGFβ1 directly, such as thrombin, neutrophil elastase or mast cell chymase may also be involved in the process of TGFβ1 activation owing to their ability to release TGFβ1 from pericellular stores [34,35].

## TGFβ1 receptors and signaling

Six receptors have been identified for TGFβ1 [36], but the most studied are the 65-KD type I receptor (Tβ RI or ALK-5), the 85-KD type II receptor (Tβ RII), the 280-KD type III receptor (Tβ RIII or betaglycan, a heparan sulfate/chondroitin sulfate proteoglycan), and more recently the 504-KD Tβ R5, which is also known as the low-density lipoprotein receptor-related protein 1 (LRP1). The canonic mechanisms by which TGFβ1 binds and activates its cognate type I and type II cell surface receptors as well as the intracellular signaling pathways that transduce TGFβ1 messages from the cell membrane to the nucleus have been reviewed extensively [10]. Briefly, TGFβ1 initially binds to the single transmembrane, constitutively active, serine/threonine kinase Tβ RII homodimer. The formed complex subsequently recruits the single transmembrane, activable, serine/threonine kinase Tβ RI homodimer, which is concomitantly activated by Tβ RII-mediated phosphorylation of several threonine and serine residues in its intracellular GS juxtamembrane domain. This phosphorylated GS domain then serves as a docking site for activin-receptor activated Smads (AR-Smads; namely Smad2 and Smad3), which are, in turn, phosphorylated by Tβ R1. The phospho-AR-Smads (pSmad2 and pSmad3) then homo or hetero-oligomerize with each other and with at least one co-mediator Smad (Co-Smad; most often called Smad4) and the complex ultimately translocates to the nucleus where it binds Smad binding element (SBE)-containing promoters or interacts with other transcriptional partners to regulate gene expression. Apart from the Smad pathway, it is now clear that other intracellular signaling pathways such as mitogen-activated protein kinase (MAPK), the phosphoinositide 3-kinase (PI3K), the PP2A phosphatase-mediated p70^S6K ^inactivation and the Rho-family of small guanosine triphosphatase (GTPase) pathways are activated by TGFβ1 and transduce some of its biological activities (reviewed in [37] and [38]). In addition, Smads were shown to cross talk with other important signaling pathways such as Janus kinase-Signal transduction and activator of transcription (JAK-STAT) [39] and WNT [40].

The overall activity of TGFβ1 can thus be regulated at different levels. Any default in proteins involved in the processes that regulate TGFβ1 expression, maturation, secretion, extracellular trafficking and localization, activation/inhibition and binding on its multiple receptors, as well as any default in receptor expression/function/distribution or in the different signaling intermediate molecules that transduce its biological effects intracellularly are susceptible to influence TGFβ1 response.

TGFβ2 and TGFβ3 also bind and signal through the same cell-surface Tβ RI and Tβ RII. Consequently, these cytokines share several biological activities *in vitro *and it is thus believed that they can substitute for each other's function *in vivo*. However, knock-outs of each of these individual genes have demonstrated non-overlapping functions of these proteins *in vivo *[41-43]. These results may stem from their different promoters, which suggests different expression patterns and regulation [44]. In addition, TGFβ2, but neither TGFβ1 nor TGFβ3, required Tβ RIII (betaglycan) to manifest its effector functions. Whether this is related to a three-amino acid divergence that affects the binding affinity to Tβ RII between the sequence of TGFβ2 and the two other isoforms [45] or the lack of RGD sequence in TGFβ2 (which is necessary for cell-surface integrin interaction and concomitant three dimensional-positioning that allows the ligand to access its cognate receptor on cellular plasmalemma) is still a contemporaneous debate, but Tβ RIII is definitely a prerequisite for TGFβ2 activity [46]. This peculiarity confers cell-specific activity to TGFβ isoforms, such that only cells expressing the Tβ RIII can response to TGFβ2, and may thus contribute to the non-redundant effects of these cytokines *in vivo*.

Disregulation of TGFβ1 activity has previously been shown to be involved in a diverse spectrum of pathologic conditions, such as cancer, autoimmunity and fibrotic diseases. The potential role for TGFβ1 in asthma pathogenesis has also been reviewed recently [44,47,48]. However, this last assertion is based on the conjecture that TGFβ is overexpressed in asthma. Unfortunately, the studies investigating the expression of TGFβ1 in asthma have yielded inconsistent results. The first purpose of the current work is to review the published data concerning the expression of TGFβ1 in asthma and to discuss the cellular sources to this cytokine in this particular disease. Secondly, recent evidence that TGFβ1 signaling is activated in the airways of asthmatics is presented and a hypothesis is proposed that TGFβ1 overactivation in asthma may not rely exclusively on its increased expression, but may be related to different alterations in other points of regulation that modulate TGFβ1 activity.

## Expression of TGFβ1 in asthma

**Figure 1:**
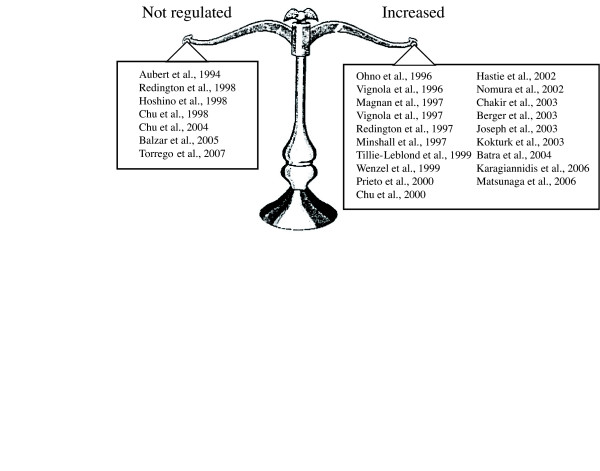
Compilation of studies investigating the expression of TGFβ expression in human asthma.

Expression of TGFβ1 is altered in asthma and the current weight of evidence suggests that TGFβ1 is upregulated in human and animal asthmatic airways (summarized in Table 1 [see additional file 1], Table 2 [see additional file 2], and Figure 1). However, 6 studies performed with human tissues have shown no regulation of TGFβ1 expression in asthma. In contrast to their previous articles, in which they reported an increased expression of TGFβ1 in bronchoalveolar lavage fluid (BALF) before and after allergic challenge [49], Redington and coworkers [32] have demonstrated indistinguishable pattern of TGFβ1 immunohistochemical staining between asthmatic and control subjects. Aubert and coworkers [50] had previously reported similar findings, but their results were contested since their control subjects were heavy smokers. The relative intensity of TGFβ1 immunostaining in the bronchial mucosa was also similar between asthmatics and healthy subjects in Hoshino and coworkers' study [51]. More recently, two papers published by the same group confirmed these results by documenting a lack of significant augmentation of TGFβ1 immunoreactivity in asthmatic epithelium [52], as well as no difference in the number of cells staining positive for TGFβ1 in the submucosa of normal subjects and asthmatic patients suffering from different severity of the disease [53].

Reasons for these discrepancies are currently unknown. However, all conflicting results came from studies measuring TGFβ1 expression by immunohistochemical approaches, using tissue specimens obtained by bronchial biopsies or lung resections. Immunohistochemistry requires extensive tissue handling. All the steps before microscopic reading, including immediate precaution to preserve tissue integrity, reagents used for fixation or to embed the tissue, strength and specificity of the detection and the staining antibodies, and bleaching of the fluorochrome or attenuated chemiluminescence signal occurring during the procedures could all lead to erroneous results and false interpretations. It is thus reasonable to surmise that the conflicting results concerning the increased expression of TGFβ1 in the airways of asthmatics may be the result of technical artefacts. However, alternative hypotheses may explain this conundrum.

### Temporal concerns

It is worth mentioning that collection of lung specimens offers conspicuous advantages for studying mRNA or protein expression at the tissue level. For example, staining of cross-sectional sections of these lung specimens by immunohistochemical approach or by *in situ *hybridization brings ample information regarding the tissue or the cellular sources of TGFβ1. Combined with laser microdissection, tissue specific expression of a particular gene can even be confirmed using more conventional techniques such as RT-PCR [54]. Unfortunately, limits of these techniques are also prominent. As such, results obtained from these experiments must be interpreted with caution. Protein or mRNA detected in lung specimens reflect their expression levels at a particular time point. Asthma is a waxing and waning disease, where a period of exacerbation is usually followed by a period of remission and where the severity of symptoms is temporally associated with the degree of airway inflammation. Therefore, upregulation of asthma mediators, such as TGFβ1, is also likely to be inducible and transient in nature. Accordingly, TGFβ1 was shown to be increased at 24 h, but not at 10 min, following segmental allergic challenge (SAC) and its concentration returned to baseline level after 1 week [49,55]. Whether TGFβ1 expression starts to increase earlier is unknown, but in animal models of acute or chronic antigen challenge, TGFβ1 expression in BALF is still unaffected 6 h following the previous allergen exposure [56]. In contrast to Batra and coworkers [55], Redington and coworkers [49] also reported a statistically significant increase in TGFβ1 level in asthmatics at baseline compared to healthy controls (8 pg/ml vs 5.5 pg/ml), but whether this difference is physiologically relevant remains questionable. Tillie-Leblond and coworkers [57] have also reported no difference in the levels of latent and active forms of TGFβ1 in BALF at baseline between mild asthmatics and healthy volunteers. In the same study, both the latent and active form of TGFβ1 were significantly increased in patients suffering from *status asthmaticus *compared to healthy controls or to patients presenting similar severity of the disease, but distant from an acute exacerbation period. Nomura and coworkers [58] have substantiated these results by examining longitudinal changes that occur in the lung function (forced expiratory volume in 1 sec, % of predicted (%FEV1)) and the percentage of TGFβ1 positive cells in induced sputum samples of five asthmatic subjects. They demonstrated that during asthma exacerbation, %FEV1 decreased from 86.5 to 51.0% and that TGFβ1 positive cells rose from 1.9 to 55.4% during the same time period. These results confirmed the inducible and transient upregulation of TGFβ1 that has been demonstrated by others in BALF following SAC [49,55]. Based on results obtained with animal models of chronic allergen challenge-induced airway remodelling, it was also suggested that several allergen provocations may be required before the upregulation of TGFβ1 could be appreciated [59].

These aformentioned findings suggest that the samples would need to be collected following bronchoprovocation to observe the transient increase in TGFβ1 expression by immunohistochemistry. In all studies documenting an absence of regulation of TGFβ1 expression in asthma, lung specimens had been taken at baseline, i.e. in a remission period where no sign of exacerbation was present or without prior experimentally-induced bronchoprovocation. In Hoshino and coworkers' study [51] for example, asthmatics presented daily symptoms, but based on their attack score, the number and severity of symptoms were very low, suggesting that subjects were not in an exacerbation period when biopsies were taken. In the immunohistochemical study carried out by Redington and coworkers [32], asthmatic subjects were presented as mildly symptomatic. However, they were clinically stable despite being restricted from use of oral or inhaled glucocorticoids for 4 weeks, indicating once again that no acute exacerbation was present at the time bronchial biopsies were taken. Hence, failure to demonstrate a significant upregulation may simply reflect the punctual expression of TGFβ1 measured in the two extreme poles of a transient response. Taken together, these results imply that TGFβ1 is not necessarily overexpressed in asthmatics at baseline, but is inducible upon allergen challenge. Determining the sequence and the kinetics of TGFβ1 expression may be important to increase our understanding of the role of this cytokine in asthma.

### Spatial concerns

In striking contrast with the results obtained by Redington and coworkers [49] and Batra and coworkers [55] in BALF, no regulation of TGFβ1 immunostaining was observed in the bronchial mucosa of asthmatics 24 h following allergen challenge [60]. This later study equally showed that the percentages of peribronchial eosinophils and neutrophils staining positive for TGFβ1 were identical following either saline or allergen challenges. Since the number of both of these cells was shown to be increased in this particular tissue after allergen challenge in their study, one might expected that if the percentage of cells expressing the cytokine remains similar, the absolute amount of TGFβ1 will be upregulated. And this will likely be reflected in the BALF, as observed by the previous groups [49,55]. It is unfortunate that the authors did not comment on this last possibility. Taken together, these results suggest that the failure to detect an increased expression of TGFβ1 in certain immunohistochemical studies may simply be related to the airway tissue studied.

Expression of TGFβ was also shown to be heterogenous within the same sample [61] and its increased expression in asthma may occur exclusively in very localized compartments. For instance, periglandular tissues or sites of epithelial desquamation were shown to stain strongly for this cytokine [61,62]. On the other hand, Magnan and coworkers [63] have demonstrated homogenous intensity of TGFβ immunostaining in ciliated and mucous cells as well as in areas of epithelial impairment, such as sites of deciliated cells or desquamated regions. However, apart from a homogenous staining in the epithelium within each sample, Magnan and coworkers [63] suggested an altered compartmentalization of TGFβ expression in asthmatic airways. Whereas TGFβ immunoreactivity was strong in the epithelium of control subjects, negative or faintly positive staining was observed in this particular compartment of asthmatics. In contrast, asthmatics expressed higher amounts of TGFβ in the submucosa compared to healthy individuals. This epithelial to submucosal redistribution of TGFβ was in accordance with an increased number of inflammatory cells staining positive for TGFβ in the submucosa of human asthmatics [61,64-68].

Whatever the physiologic or pathophysiologic reason for this altered compartmentalization, the same trend of TGFβ1 relocalization was observed in murine models of allergic airway inflammation. In this regard, McMillan and coworkers [69] have demonstrated that TGFβ1 expression was confined to the bronchiolar and alveolar epitheliums in control animals and was relocalized to the submucosal compartment in association with inflammatory infiltrates after repeated allergen challenges of sensitized animals. In this particular model, even smooth muscle became positive for TGFβ1 immunostaining during the chronic phase of allergen challenge. Interestingly, this altered compartmentalization also occurred in other types of airway inflammation, such as the one induced by prolonged (4 wk) lipopolysaccharide (LPS) exposure [70]. Initially, TGFβ1 expression was confined to the airway epithelium, but subsequent to LPS exposure, TGFβ1 immunostaining was mainly localised in the subepithelium area [70]. Hence, in addition to looking at the right time, investigators attempting to document an increased expression of TGFβ1 in asthma need to look at the right place.

With the use of techniques permitting to appreciate the overall expression of TGFβ1, such as in studies using BALF, serum or plasma, or with the use of animal models, which allow sufficient biologic materials to be homogenized, it is becoming clear that TGFβ1 is upregulated in asthma following allergic challenge. But once again, controversies are reported and are related to different peculiarities of the studied populations. For example, Joseph and coworkers [71] have reported an increase in TGFβ1 expression in the plasma of nonatopic, but not of atopic asthmatic patients. However, using only atopic patients, which were included based on skin prick test positivity and corroborating medical history of allergen-induced asthma, Karagiannidis and coworkers [72] reported a significant increase of TGFβ1 in the serum of asthmatics, attaining levels almost 7-fold higher than those measured in healthy controls. No clear explanation is currently proposed to explain these contrasting results and the question of whether TGFβ1 expression is influenced by the atopic status will need further exploration.

Increased expression of TGFβ1 measured in the BALF must also be interpreted with caution. Epithelium desquamation is a characteristic feature of remodeled asthmatic airways. Epithelium denudation may give access to a certain amount of TGFβ1, which is otherwise masked by an intact epithelium in non-asthmatic individuals. Thus, the increased expression of TGFβ1 observed in BALF of asthmatics following challenge may simply be related to an easier accessibility to TGFβ1 stores cause by epithelium desquamation. In support of this contention, increased concentration of TGFβ1 has been noted in BALF following a sham bronchoprovocation procedure, which is likely to be the result of epithelial damage [49]. Moreover, a positive correlation (r = 0.89) has been reported in the same study between concentration of TGFβ1 and the number of epithelial cells collected in BALF of the saline-challenged site. These results suggest that epithelium denudation renders a bulk of TGFβ1 normally sequestered in the subepithelial layer, such as the one associated with the basal lamina [32,50], collectable by bronchoalveolar lavage. Of major concern, degranulation products of eosinophils such as major basic protein (MBP) and eosinophil cationic protein (ECP) [73,74], as well as mast cell proteases, such as tryptase and chymase [75], are damaging for the airway epithelium. Increased expression of TGFβ1 observed at 24 h, but not at 10 min, following SAC may consequently be caused by eosinophil- or mast cell-mediated epithelium desquamation, rather then a true *de novo *protein synthesis of TGFβ1. Together, these observations raise doubts on the techniques currently used to measure the expression of different mediators in the airways and, for instance, the increased expression of TGFβ1 in asthma.

## Cellular sources of TGFβ1 in asthma

Studies investigating the cellular source of TGFβ1 in asthma have also yielded inconsistent results. It is known that TGFβ1 is widely expressed throughout the body and that every resident structural and immune cell in the lung, as well as every inflammatory cell mobilised to the airways during asthma exacerbation, is able to express and secrete TGFβ1. In the lungs of non-asthmatic humans or animals, airway epithelium seems to be the major site of TGFβ1 expression [16,63,76-80]. However, other stromal cells in the airways such as fibroblasts [61,81-83], endothelial cells [84], vascular smooth muscle (VSM) cells [78] and airway smooth muscle (ASM) cells [50,62,77,78,85-90] are also potential source of this cytokine since they were all shown to express and produce detectable amount of TGFβ1. Due to its affinity to certain components of the ECM, latent forms of TGFβ1 in the lung have a tendency to accumulate in particular compartments of the airway wall. In fact, many immunohistochemical studies have localized TGFβ1 mainly in extracellular compartments in association with connective tissues of the airway wall [32,50,62]. However, the cellular sources of ECM-sequestered TGFβ1 are difficult to determine. In contrast to these results, Magnan and coworkers [63] were unable to identify TGFβ expression in extracellular space, but rather identified inflammatory cells infiltrating the submucosa and the epithelium as the major source of this cytokine. One could speculate that this controversy may be related to the use of a pan-TGFβ antibody (Ab) in this latter study, but the detection of all three forms of TGFβ instead of TGFβ1 only would be an additional reason to find TGFβ in extracellular spaces.

### Neutrophils

In asthma, the cellular origin of TGFβ1 is less clear and numerous inflammatory cells as well as structural cells were shown to contribute. Blood- or airway-derived neutrophils in normal and asthmatic individuals were shown to express TGFβ [60,66,91]. Since, airway neutrophilia is particularly prominent in nonatopic asthma [92] and in more severe forms of the disease (reviewed in [93]), neutrophils could contribute significantly to the increased expression of TGFβ in these types of asthma. In this regard, Chu and coworkers [66] have demonstrated that around 55% of TGFβ-positive cells in the submucosal compartment of asthmatics and normal controls were neutrophils [66]. However, only a fraction of neutrophils expressed TGFβ (29 and 20% in asthmatic and normal submucosa, respectively). In animals, increased TGFβ1 expression in the subepithelial area following prolonged (4 weeks) exposure to LPS was also neutrophil-dependent, as neutropenic animals did not develop this altered expression of TGFβ1 [70]. Interestingly, upregulation of TGFβ1 expression in the airway epithelium observed after a recovery period of 4 weeks following exposure to LPS was also dependent on the presence of neutrophils during the LPS exposure period [70]. Hence, it was concluded that neutrophils may be a direct source of TGFβ1 following their mobilisation into the submucosa, but may also alter the subsequent expression of TGFβ1 in other airway compartments, for instance the epithelium. In addition to the epithelium, Lee and coworkers [89] have recently demonstrated that neutrophil elastase increased the expression of TGFβ1 in ASM cells, suggesting once again that the mobilisation and activation of neutrophils into the airways may increase TGFβ1 via indirect mechanisms. Since TGFβ1 is also recognised as a potent trophic factor for granulopoiesis [94] and was shown to recruit, activate and prolong survival of neutrophils in other diseases [95], TGFβ1 upregulation and neutrophilia observed in asthma may mutually feedback each other, allowing the establishment of a vicious cycle potentially involved in disease exacerbation.

### Eosinophils

In contrast to the aforementioned studies, Ohno and coworkers [64] claimed that close to 100% of cells positive for TGFβ1 mRNA in mild and severe asthma were eosinophils, whereas these cells accounted for only 20.8% of total TGFβ1 mRNA-positive cells in control subjects. Similarly, Flood-Page and coworkers [67] have shown that 86% of cells positive for TGFβ1 mRNA in the bronchial mucosa were eosinophils, and that 76% of the total eosinophil population in this tissue compartment was immunolabeled. These findings were substantiated by Minshall and coworkers [65], who reported a coefficient of determination (R^2^) of 0.86 between TGFβ1 mRNA-positive cells beneath the basement membrane and the degree of eosinophilia in the same compartment. In this latter study, 65% of the TGFβ1 mRNA positive cells were eosinophils and 75% of eosinophils were positive for TGFβ1 mRNA. The remaining TGFβ1 mRNA positive cells were identified as macrophages and fibroblasts [65]. Finally, Vignola and coworkers [61] reported that eosinophils accounted for 80% of TGFβ1 mRNA positive cells in the submucosa, the other 20% being fibroblasts.

As reported by Minshall and coworkers [65], many other studies performed in humans or in animals bear witness to the contention that eosinophils are a major cellular source of TGFβ1 in asthmatic lungs by demonstrating a linear relationship between the degree of eosinophilia and the expression of TGFβ1 in different lung tissues. For example, Nomura and coworkers [58] found a positive correlation between eosinophil counts and the number of cells staining positive for TGFβ1 in induced sputum samples of asthmatics. In a murine model of allergic asthma, Tanaka and coworkers [96] reported a positive correlation between the number of eosinophils and the level of TGFβ1 in whole-lung lavage fluid. It was also proposed that eosinophils from bronchial asthmatics are more competent in TGFβ1 secretion compared to eosinophils of control subjects, owing to their elevated TGFβ1 mRNA [65] and protein [97] levels per cell.

In inflammatory conditions affecting the upper airways, such as nasal polyps or allergic rhinitis, the cells expressing TGFβ1 gene in the mucosa specimens were also identified as being predominantly eosinophils [97]. The proportion of eosinophils positive for TGFβ1 gene was estimated at 50%. However, a great deal of TGFβ1 protein in these specimens was not cell-associated, but rather localized in the ECM associated with the vessels, the basement membrane or within the submucosa [97]. This latter result was consistent with several immunohistochemical studies investigating the expression of TGFβ1 in lung specimens [32,50,62].

IL-5 knockout mice also support the role for eosinophils in TGFβ1 production. Compared to wild type animals, IL-5-deficient mice chronically exposed to allergic challenge showed a decreased expression of MBP positive cells, which paralleled the reduction in the number of TGFβ positive cells in the peribronchial region and a decrease in TGFβ1 expression in whole lungs [98]. These changes in TGFβ1 expression also correlated with fewer signs of airway remodeling. In accordance with this animal model, treatment of mild atopic asthmatics for 2 months with anti-IL-5 Ab (mepolizumab) was shown to be successful in reducing tissue eosinophilia, lung TGFβ1 expression and deposition of ECM components in the lamina reticularis [67]. Therefore, the fact that both IL-5-deficient mice [98] and asthmatics treated with anti-IL-5 Ab [67] demonstrated fewer eosinophils, less TGFβ1 expression and fewer signs of remodeling supports the notion that eosinophils are an important source of TGFβ1 in the lungs of asthmatics and that IL-5-dependent recruitment of eosinophils is a prerequisite for TGFβ1-mediated airway remodeling. However, it is worthy of mention that the statistically significant decrease in the median level of TGFβ1 in BALF reported by Flood-Page and coworkers [67] following anti-IL-5 Ab treatment was approximately 1 pg/ml, which is unlikely to be physiologically relevant.

### Macrophages

In the above study with IL-5-deficient mice, Cho and coworkers [98] also demonstrated that 35% of TGFβ1 positive cells in the peribronchial region were macrophages. In humans, macrophages recovered in induced sputum samples also expressed TGFβ1 [58] and alveolar macrophages from asthmatics released spontaneously higher amounts of TGFβ1 relative to alveolar macrophages derived from control subjects [99]. Similarly, Prieto and coworkers [100] found higher level of TGFβ1 mRNA in alveolar macrophages of mild atopic asthmatics compared to healthy subjects. In Magnan and coworkers' study [63], TGFβ positive cells present in the submucosa of asthmatics were mainly identified as lymphocytes, macrophages and, to a lesser extent, eosinophils. In a transgenic model of asthma induced by lung overexpression of IL-13, TGFβ1 mRNA and protein were observed mainly in macrophages, but also in type II pneumocytes, airway epithelial cells and occasionally in eosinophils [16]. In a mouse model of prolonged allergen challenge-induced airway remodeling, the main source of TGFβ1 was identified as mononucleated cells, likely macrophages [69]. Collectively, these studies suggest that macrophages are a likely source of TGFβ1 in asthma.

The higher levels of TGFβ1 mRNA in BALF-derived alveolar macrophages (AM) observed by Prieto and coworkers [100] in mild atopic asthmatics at baseline was not further increased following repeated low-dose allergen inhalation. Similarly, the increased release of TGFβ1 by alveolar macrophages derived from asthmatics demonstrated by Vignola and coworkers [99] was at baseline (i.e. without prior allergen bronchoprovocation). These results suggest that alveolar macrophages of asthmatics produce higher amounts of TGFβ1 spontaneously. It equally raises the possibility that the baseline overexpression of TGFβ1 observed in asthma by certain investigators [62,101] is related to the increased production of this cytokine by alveolar macrophages. The teleologic advantage of an increased TGFβ1 production by these cells in asthma is unknown. However, it is worth mentioning that alveolar macrophages are predominant in the airways compared to other cells mobilised into the airways following allergen challenge or at baseline. Consequently, the mediators they produce are susceptible to influence to a great extent the pathologic outcomes. Of interest, alveolar macrophages were shown to be protective against asthma development in a rat model of asthma [102]. Owing to the well known immunosuppressive activity of TGFβ1, it is tempting to speculate that the increased expression of TGFβ1 observed in alveolar macrophages of asthmatics at baseline may represent a regulatory mechanism to mitigate the variable chronic ongoing inflammation during the stable phase of the disease. In addition, and in contrast to other airway cells, expression of TGFβ1 may not be altered in macrophages following allergen challenge.

### Mast cells

Unfortunately, the contribution of mast cells to the upregulation of TGFβ1 expression in asthma is not clear either. Based on a study using mast cell-deficient mice (W/W^v ^and Sl/Sl^d^), Masuda and coworkers [103] have demonstrated that the overall contribution of mast cells to the upregulation of TGFβ1 expression in BALF of sensitized and challenged mice was negligible. However, several groups have demonstrated that mast cells were capable of secreting TGFβ1 constitutively or upon stimulations in *in vitro *conditions [104-106].

### Epithelium

In contrast to Magnan and coworkers [63] who reported a decreased expression of TGFβ in the epithelium of asthmatics, some studies have pointed toward this tissue to explain the increased expression of TGFβ1 in the airways of asthmatic individuals [61,107]. Vignola and coworkers [61] reported that TGFβ was faintly expressed in the airway epithelium of control subjects and was significantly elevated in asthmatic subjects. Torrego and coworkers [60] did not compared asthmatic and healthy control individuals, but still agreed with this finding by showing that the main cellular source of TGFβ1 in the bronchial mucosa of mild atopic asthmatics was found in the epithelium following either saline or allergen challenges. It was also demonstrated that the spontaneous *ex vivo *release of TGFβ1 was higher in airway epithelial cells derived from asthmatic subjects compared to that derived from non-asthmatic subjects [107]. In a model of sensitized mice chronically challenged by inhalation of low doses of antigen, Kumar and coworkers [56] have demonstrated that TGFβ1 expression increased in airway epithelial cells, but not in eosinophils or any other non-epithelial cells. In addition, using laser capture microdissection and real-time PCR to quantify TGFβ1 mRNA levels in lung sections of mice, Kelly and coworkers [54] demonstrated that the TGFβ1 mRNA upregulation observed 2 wk after chronic allergen exposure in sensitized animals was confined to the airway epithelium.

Kumar and coworkers [56] have also shown that TGFβ1 in airway epithelial cells of naïve animals is in its uncleaved, biologically inactive form. Following chronic challenge of sensitized mice with low doses of antigen, the cleaved and biologically active form of TGFβ1 was found mainly in the subepithelial zone in association with connective tissue [56]. They suggested that the increased expression of TGFβ1 in the subepithelial zone simply reflects deposition of epithelial cell-derived TGFβ1 onto the subjacent ECM following its activation by antigen challenge. Hence, the concept that the increased expression of TGFβ1 in the submucosa originates from inflammatory cell infiltrates makes no unanimous consensus among investigators in the field. This result was consistent with observations made by Kokturk and coworkers [62] on human tissues, which confirmed the increased expression of TGFβ1 in the airway submucosa of asthmatics despite the lack of a simultaneous alteration in inflammatory cell infiltrate. However, only asthmatics that were free from symptoms for at least a month preceding the biopsy were included in this latter study. It is thus possible that remnant (i.e. non-utilised) inflammatory cell-derived TGFβ1 was stored in the ECM after secretion and, as a result, would be responsible for the increased TGFβ1 expression observed at a time when cellular inflammation was resolved. Otherwise, increased TGFβ1 expression may be produced by airway structural cells present in the submucosa such as (myo)fibroblasts or ASM cells [62,69].

Kumar and coworkers [56] also suggested that the concentration of antigen and the number of antigen exposures are key elements determining which cells will preferentially produce TGFβ1 in allergic asthma. They concluded that eosinophils are the main TGFβ1-producing cells in acute models of allergic asthma challenged with high doses of antigen, but the epithelium is the main source of this cytokine in sensitized animals chronically challenged with low doses of antigen.

This increased TGFβ1 production by the airway epithelium in asthma is consistent with numerous reports suggesting that epithelial cell-derived TGFβ1 could be upregulated upon different phlogogenic challenges *in vitro *[80,107-110], albeit conflicting results have been reported [111]. *Ex vivo *cultures of bronchiolar epithelial cells derived from smokers and from patients with COPD also secrete higher amounts of TGFβ compared to those of control patients [112]. In addition, TGFβ1 upregulation in airway epithelial cells occurs by mechanical stress that mimics bronchocontriction [113], as well as in several *in vivo *conditions in addition to the aforementioned animal models of asthma [54,56,98], including IL-13 transgenic mice [16], and advanced pulmonary fibrosis [114] and COPD [112] in humans.

The decreased expression of TGFβ in the airway epithelium of asthmatics reported in some studies [63] may reflect an active secretion of TGFβ. In this case, intracellular stores found in non-asthmatic epithelium would give higher staining intensity for TGFβ by immunohistochemistry, but the latter would be reduced in asthmatics as soon as the intracellular stores are emptied during the course of the disease. Physiologically, this active TGFβ secretion may be interpreted as an attempt by the epithelium to buffer excessive ongoing inflammation. Alternatively, a decreased immunoreactivity may represent a real decrease in *de novo *synthesis of TGFβ1 by the asthmatic epithelium. In this case, it may represent a well-regulated process that favours inception or perennialization of airway inflammation.

### Airway smooth muscle

The expression of TGFβ1 in ASM as been recognized for a while [50,62,77,78,85-90], but studies that have compared the expression between asthmatic and non-asthmatic individuals are limited. Berger and coworkers [88] were the first to demonstrate an increased expression of TGFβ1 in ASM layer of persitent asthmatics compared to non-asthmatic controls. More recently, Xie and coworkers used laser capture microdissection to isolate ASM tissue from bronchial biopsies, and have demonstrated that ASM from asthmatic patients expressed higher mRNA and protein amount of TGFβ1 [90]. These findings are consistent with *in vitro *results showing that different stresses that may be encountered by ASM in asthma, such as wounding [87], neutrophil elastase [89], tryptase [88] and angiotensin II [86], trigger an increased TGFβ1 expression by ASM cells. It was also reported that the chemotactic activity of ASM supernatant toward mast cells is related mainly to TGFβ1 [88]. Together with the reported possitive correlation between the levels of TGFβ1 expression and the number of mast cells in the ASM tissue of an asthmatic population [88], this ASM cell-derived, TGFβ1-mediated mast cell migration may thus be involved in mast cell myositis [115], which is a characteristic phenotypic feature of asthma [116].

Describing the pleiotropic functions of TGFβ1 on ASM cells is beyond the scope of the present review. However, it is well-established that in addition to being secreted by ASM cell *per se*, this cytokine can, in turn, bind ASM cells and influence their behavior in a way that can be extremely relevant to further our understanding of asthma pathogenesis [90,117-121].

### Cellular sources of TGFβ1 in other types of airway inflammation

In other types of airway inflammation mediated by allergen-independent mechanisms, such as the one induced by prolonged LPS exposure, TGFβ1 also increased in whole-lung lavage, as well as in the epithelium and the submucosal compartments of the lung [122,123]. Similarly, a single intratracheal delivery of an adenoviral vector containing the proinflammatory cytokine IL-1β was sufficient to increase the expression of TGFβ1 [124]. Together, these findings suggest that TGFβ1 is induced downstream of many causes of inflammation, probably acting as a counterregulatory cytokine to resolve inflammation and to initiate repair processes. If this conjecture is true, and because asthma is an inflammatory condition of the airways, it is expected that TGFβ1 would be upregulated in asthmatic airways at a later time-point following challenge. In addition, since it is a cytokine ubiquitously expressed, its cellular source in a particular disease may originate from the cells triggered by the inflammatory signals or by the inflammatory cells mobilised to the site of inflammation *per se*. Consequently, in the case of severe asthma where neutrophils predominate, neutrophils would be the main source of TGFβ1; and in the case of mild to moderate asthma where eosinophils predominate, eosinophils would be the principal cells secreting this cytokine. This hypothesis would reconcile many of the conflicting results published so far and simply suggest that TGFβ1 is upregulated as a general mechanism to circumvent inflammation and its secretion is ensured by any cells present at the site of inflammation. Therefore, inconsistencies surrounding the cellular source of TGFβ1 expression in asthma may be related to either the heterogeneity of the asthma groups studied or to the particular states of the disease (exacerbation vs remission period) when the biopsies were taken.

Some weaknesses in the studies involved in the controversial issue concerning the cellular source of TGFβ1 in asthma are also worthy of mention: Firstly, the Ab used in Magnan and coworkers [63], Vignola and coworkers [61], and Chu and coworkers [66] did not discriminate between the 3 isoforms of TGFβ and thus, the staining distribution and intensity is additionally confounded by TGFβ2 and TGFβ3 expression. Secondly, the discrepancy may also be related to the control group of Magnan and coworkers [63], half of which were smokers and all showed existing or suspected lung disease. Finally, absence of medication withdrawal in the asthmatic group before tissue collections in this same study could also have led to erroneous results.

## Active TGFβ signaling in asthma

Active TGFβ signaling, measured by nuclear immunostaining of phosphorylated Smad2 (pSmad2), has been observed in airways of animal [125-127] and human [128,129] asthmatics before and after allergic challenge. Recent observations made by Torrego and coworkers [60] further corroborates these results by showing an increased nuclear staining of Smad4, together with an increased expression of nuclear Smad2/3, in the bronchial mucosa of mild atopic asthmatics 24 h post-allergen challenge. Whether these observations are the result of an increased expression of one or many of the TGFβ isoforms, their desequestration from ECM or simply their activation could be debated, but active signaling surely testifies that one or several of these processes are operational in asthma.

However, it is noteworthy to mention that this increased pSmad2 observed in asthma does not exclude the possible involvement of other TGFβ family members in the activation of AR-Smads (Smad2/3). Activin A, in particular, has recently gained interest in asthma pathophysiology. Both mRNA levels in total lung [72] and BALF concentrations [130] of Activin β A were upregulated following OVA sensitization and challenge in mice. In humans, serum levels of Activin A were shown to be elevated in moderate asthmatics (1.16 ng/ml) compared to healthy individuals (0.14 ng/ml) [72]. These studies suggest that increased expression of pSmad2 following SAC may not be entirely related to TGFβ1, but may also be due to other cytokines of the TGFβ superfamily that signal via the AR-Smads, such as Activin A. However, the serum levels of TGFβ1 is more than 20-fold higher than those of Activin A [72]. In addition, the latter is 10-fold less potent than TGFβ1 in activating the Smad2/3 complex, as measured by transfection of human lung fibroblasts (IMR-90) with a Smad2/3-responsive reporter gene [72]. It is thus believed that TGFβ1 might outweigh the effect of Activin A, and consequently, may represent the main contributor to Smad nuclear translocation following allergen challenge.

TGFβ2 is also of particular interest in asthma. Two studies that failed to identify an increased expression of TGFβ1 in asthmatic airways have looked at TGFβ2 expression, and both revealed significant increases [52,53,60]. In one of these studies, Chu and coworkers [52] demonstrated a higher level of TGFβ2 in the airway epithelium of asthmatics compared to normal subjects. They further demonstrated that TGFβ2, but not TGFβ1, is increased in primary cultures of bronchial epithelial cells following IL-13 stimulation. This result was supported by two previous articles, in which both IL-13 and IL-4 increased TGFβ2 production in bronchial epithelial cells [109,131]. In the second study, Balzar and coworkers [53] quantified the number of cells staining positive for TGFβ in the submucosa. They demonstrated that among the 3 TGFβ isoforms, only TGFβ2 was increased in asthmatics. This increased expression was also restricted to the group of patients demonstrating the more severe form of the disease with persistent eosinophilia, which is surprisingly similar to the finding published earlier by the same group using a pan-TGFβ Ab [132]. Moreover, they showed that tissue eosinophils from the severe group of patients expressed higher amounts of TGFβ2 compared to tissue eosinophils of control subjects or from patients suffering from a milder form of the disease. Hence, in addition to the increased production of TGFβ2 by the airway epithelium in response to T_H_2 cytokines [52,109,131], eosinophils in the submucosa could contribute to the overall increase of TGFβ2 in the airways of severe asthmatics [53].

Elevated expression of TGFβ2 following bronchoprovocation was also demonstrated in two recent papers [55,60]. In these studies, TGFβ2 expression was increased in BALF [55] and in the bronchial mucosa [60] 24 h after allergen challenge. However, Batra and coworkers [55] also noted a higher expression of TGFβ2 in non-asthmatics at baseline as well as 1 and 2 weeks after SAC compared to asthmatic subjects. In fact, only at 24 h post-SAC did TGFβ2 levels in BALF of asthmatics reach the concentration found in non-asthmatics. Collectively, these results suggest that TGFβ2 is increased in the airway epithelium of asthmatics [52], as well as in eosinophils of a subgroup of severe asthmatics [53], and even if its baseline expression in the fluid harvested by bronchoalveolar lavage is lower compared to non-asthmatics, it is transiently increased after allergen challenge [55]. Since the TGFβ2 isoform acts on the same receptors and signal via the same AR-Smads as TGFβ1, these results indicate that TGFβ2 is also a likely candidate to explain activation of Smad signaling in asthma.

However, antibody to TGFβ1 was shown to prevent phosphorylation of Smad2 in a murine model of prolonged allergen challenge-induced asthma [126]. This finding suggested that TGFβ1 is responsible for the increased expression of pSmad2 in the airways of asthmatics and excluded the possible involvement of TGFβ2 or Activin A. Interestingly, this anti-TGFβ1 Ab was administered following the establishment of eosinophilic inflammation, and in addition to abrogating pSmad2 signaling *in situ*, it reduced total and proliferating ASM cell numbers, mucus production and peribronchiolar ECM deposition. These results suggest that anti-TGFβ1 therapy can be envisaged as a therapeutic approach (i.e. following the establishment and the diagnosis of the disease), not only to reverse fibrosis, but also to alleviate other features of airway disease in asthma.

In addition to active TGFβ signaling demonstrated by others [125-129], Leung and coworkers [133] showed an increased expression of Smad2/3 in ASM tissue of Brown Norway rats sensitized and exposed to allergen. This result indicates that the asthmatic state may prime ASM tissue to respond in an excessive manner to TGFβ. This altered expression of Smad2/3 was abrogated with oral administration of SD-208, a pharmacological inhibitor targeting ALK5, which suggests that TGFβ is involved in the upregulation of its own signaling intermediates that ensure its signal transduction. Interestingly, both preventive and curative treatments with SD-208 successfully abrogated ASM cell hyperplasia. These treatments equally reduced airway inflammation and goblet cell hyperplasia, suggesting a potent pro-inflammatory action of TGFβ in asthma in addition to its well-recognized function in airway remodeling. These finding also highlight, once again, the beneficial effect of a strategy preventing TGFβ signaling in reversing established features of airway disease.

The anti-inflammatory effects observed with SD-208 are counterintuitive to the well-known immunomodulatory function of TGFβ1 [133]. In fact, several pieces of evidence have shown that TGFβ1 counteracts excessive airway inflammation. Examples include the following: 1-TGFβ1 heterozygous mice, which express 30% of the TGFβ1 protein level observed in the wild type animal, develop a more severe form of the disease when exposed to an OVA sensitization/challenge protocol [134]; 2- T lymphocytes engineered to produce TGFβ1 or conditioned to secrete higher amounts of TGFβ by oral tolerance reverse and ameliorate, respectively, allergen-induced airway inflammation [135,136]; and 3- blocking TGFβ signaling in T cells by overexpressing Smad7 enhances allergen-induced airway inflammation [129]. However, the findings obtained with SD-208 indicate that considering TGFβ1 only as an immunosuppressive cytokine can be misleading. In support of the inflammatory role of TGFβ1 in asthma, others have shown that its release by structural cells in the airways contributes to inflammatory cell recruitment [88,115]. In fact, TGFβ1 is a powerful chemotactic factor for monocytes/macrophages [137], eosinophils [138], neutrophils [139] and mast cells [140]*in vitro*. Migration of monocytes/macrophages toward a gradient of TGFβ1 occurs at concentrations in the femtomolar range [137]. Additionally, TGFβ1 has been shown to rescue murine macrophages from apoptosis [141]. *In vivo*, the number of mast cells in ASM bundles was positively associated with the ASM tissue expression of TGFβ1 [88]. Given the important function of mast cells in the pathophysiology of allergic asthma [116], increased secretion of TGFβ1 by structural cells in the airways following allergic challenge may foster, rather than attenuate, inflammation.

## Speculative argument

With all data taken together, one might imagine the following scenario of TGFβ1 regulation in asthma and its potential role in the pathogenesis of the disease. In the first stage of the disease, structural cell-derived TGFβ1 may be released, or simply activated, to induce tissue infiltration of antigen presenting cells (APC, i.e. monocytes and dentritic cells) and mast cells. Both of these cell types are required for an immunologic response to take place in the airways. On one hand, APC capture and process the allergen and then migrate to regional lymph nodes to build a T- and B-lymphocyte immunologic response. On the other hand, mast cells home to the airway walls and will bind to B-cell-derived IgE to produce an allergen-specific reaction upon subsequent allergen exposure. In this scenario, it is thus inferred that TGFβ1 is implicated in the inception of allergic asthma by fostering the sensitization process. In later stages of the disease (i.e. in already sensitized individuals), TGFβ1 synthesis by structural cells may stay downregulated for a while to favor the establishment of lymphocytic and neutrophilic/eosinophilic inflammation. These mobilised inflammatory cells are first programmed to synthesize or secrete pro-inflammatory mediators and to sequentially express and secrete immunosuppressive cytokines, such as TGFβ1, to prevent excessive inflammation and damage. At later time points, when the bulk of inflammation is resolved, alveolar macrophages would maintain their higher secretion of TGFβ1 and the airway epithelium would start to express TGFβ1 again [54]. Increased expression of TGFβ1 by both of these cellular sources may aim to get rid of remnant inflammation and to pursue the healing response. In this scenario, when TGFβ1 action is well regulated, restitution of airway wall integrity would take place and airway function would be recovered. Otherwise, when TGFβ1 actions are uncontrolled, airway remodeling would likely occur.

## TGFβ1 receptors in asthma

TGFβ receptors are also expressed ubiquitously on mammalian cells [142-145]. In the lung, Tβ RI and Tβ RII were identified in macrophages, as well as in epithelial, VSM, ASM and endothelial cells of both conducting airways and alveoli [78]. In contrast to its ligands, only few studies have documented the regulation of these receptors in asthmatic airways. Balzar and coworkers [53] have demonstrated that Tβ R1 is downregulated in mild and severe asthma. Similarly, Barbato and coworkers [146] have reported a decrease in the number of cells positive for Tβ RII in the subepithelium of children with asthma. According to the authors, these results may be indicative of active TGFβ signaling, which is associated with TGFβ receptor internalization. In contrast, wounding [119] as well as granulocyte-macrophage colony-stimulating factor (GM-CSF) treatment [147] have been shown to increase TGFβ receptor expression in monocultures of ASM cells. Since ASM cells are subjected to different kinds of damaging stress and that GM-CSF is upregulated in the lungs of asthmatic patients [61,148,149], these *in vitro *observations will require further attention as they may actually subtantiate the biological effect of TGFβ1 on ASM cells *in vivo*.

## Conclusions and perspectives

Despite being extensively studied, the expression of TGFβ1 in the airways of asthmatics still elicits more questions than answers. The current weight of evidence suggests that TGFβ1 is upregulated in asthma. However, whether its expression is altered at baseline or it is upregulated in a transient fashion following bronchoprovocation is not clear and may depend on the particular cell and/or tissue studied. For instance, baseline expression of TGFβ1 seems to be increased in alveolar macrophages [99,100], but its expression in the airway lumen appears to be inducible. In support to the latter contention, two groups of investigators that have collected BALF following SAC in human asthmatics have demonstrated that the TGFβ1 level is transiently increased [49,55,150]. On the other hand, the kinetics of TGFβ1 expression in the airway epithelium of asthmatics seem to be more complex since both upregulation [61,107] and downregulation [63] have been reported. Studies looking at the kinetics of TGFβ1 expression in this particular tissue following allergen challenge should shed light on these conflicting results.

As pointed out in this review, all the studies that were unable to detect an altered protein expression of TGFβ1 in asthma were investigating its expression levels by immunohistochemistry. However, it is also important to mention that certain investigators were capable to demonstrate an increased expression of TGFβ1 in asthmatic airways at baseline (i.e. without prior allergen challenge or apparent asthma exacerbation) by using immunohistochemistry. For example, Minshall [65], Chakir [68], Kokturk [62], Berger [88] and Xie [90] with their respective coworkers have all previously shown an increased intensity of staining or an increased number of positive cells for TGFβ1 in the subepithelial region of asthmatic airways. In most cases, it seems to be associated with the severity of the disease [65,68,88] and the immunohistochemical signal seems to originate from inflammatory cells infiltrating the submucosa [65,68] or ASM [88,90]. Owing to the static picture obtained by staining lung sections by immunohistochemical techniques and because endobronchial biopsies in humans are performed when the patients are free of symptoms, the lack of increased expression of TGFβ1 reported by these groups of investigators can reflect the failure to capture the transient TGFβ1 upregulation. This may be the reason why more immunohistochemical studies seem to be involved in the controversial issue highlighted in this review. Alternatively, reported inconsistencies may be the result of technical artefacts related to immunohistochemistry or to inherent heterogeneity of asthma pathogenesis among different populations.

Limitations with BALF procedure were also highlighted in this review and it may thus be too soon to reject the possibility that TGFβ1 in not overexpressed in asthma. Studies designed to harvest tissues at multiple-time points following allergic challenge would be very useful to understand the kinetics of this cytokine regulation. Considering the invasive nature of trans- or endo-bronchial biopsies or BALF, repeated measurements seem quite unrealistic in human subjects. Less invasive techniques of investigation, such as induced sputum [58,151] or exhaled breath condensate [101], represent interesting alternatives for these multiple time point studies and have previously been used successfully to assess TGFβ1 expression in human subjects. The question that remains is to what extent these techniques accurately reflect TGFβ1 expression in deeper airways. As such, the validity of these techniques needs to be tested. Otherwise, identification of other surrogates of lung TGFβ1 expression that could be readily measured by these less invasive techniques would be required. A last possibility to understand the kinetics of TGFβ1 expression and activation would be to consider the use of animal models.

In the meantime, increased expression of TGFβ1 has been shown to occur exclusively in restricted localization in the airways or only in particular subgroups of patients, such as in severe asthmatics demonstrating prominent eosinophilic inflammation [132]. Hence, in addition to temporal concerns, spatial concerns need to be considered. The fact that TGFβ1 may be differently involved in the pathogenesis (or in the remission) of asthma in phenotypically distinct groups of asthmatics must also be appreciated. In addition, a debate still persists concerning the inflammatory cell that is mainly involved in the generation of TGFβ1 in asthma, as eosinophils, macrophages, neutrophils, epithelial cells and ASM cells have all been pointed out. Whether these conflicting results mirror the heterogeneity of the disease in term of triggering agents, individual genetic variability, history and severity of the disease, or whether they are simply related to the time points or the tissue chosen to measure TGFβ1 expression are still unresolved questions and will required further explorations.

Since TGFβ1 is released in an inactive form, its kinetics of activation will also be relevant to elucidate its biological or pathobiological functions in asthma. As highlighted in this review, several points of regulation can influence the final magnitude of the TGFβ1 response. All of these points of regulation are as important as the expression of TGFβ1 *per se *if one attempts to appreciate the overall contribution of this cytokine in airway pathogenesis that characterizes asthma. Unfortunately, studies investigating these points of control are limited. On the other hand, several studies indicate that TGFβ1 activity is increased in the airways of asthmatics, as measured by intermediary end points of TGFβ1 signaling (pSmads), which indicate that TGFβ1 has been activated and has bound to its cognate cell-surface receptor. These results suggest that if TGFβ1 is not upregulated in asthma, other points of control must be altered and this is ultimately translated into an increased TGFβ1 activity. However, these results do not exclude the involvement of other TGFβ superfamily members that signal via the same Smads. But in this regard, one study suggested that among these family members that demonstrated an upregulated expression in asthma, TGFβ1 is likely the main contributor to the increased Smad signaling [126].

Dissecting the kinetics of TGFβ1 regulation following bronchoprovocation will undoubtedly increase our understanding of the inflammatory and fibrotic processes that take place in asthma. Due to its large spectrum of biologic effects, it is also unfortunately too soon to determine whether TGFβ1 is the good guy or the nasty guy in asthma. However, results obtained recently with anti-TGFβ1 Ab [126] or with an ALK-5 inhibitor (SD-208) [133] in animal models of the disease are promising and suggest that targeting TGFβ1 may be beneficial in the treatment of human asthma.

## Competing interests

The author(s) declare that they have no competing interests.

## Supplementary Material

Additional file 1 Expression of TGFβ1 in human asthma. The table provided is a compilation of all the studies published so far regarding the expression of TGFβ1 in different lung compartments of human asthmatics. Click here for file

Additional file 2 Increased expression of TGFβ1 in animal models of asthma. The table provided is a compilation of published studies regarding the expression of TGFβ1 in different lung compartments of animal models of asthma. Click here for file
