# Supplementary material for: Controversy surrounding the increased expression of TGFβ1 in asthma
Source: Respir Res. 2007 Sep 24;8(1):66. doi: 10.1186/1465-9921-8-66 (PMC2078588; doi:10.1186/1465-9921-8-66)
Supplement: Additional file 1 — Expression of TGFβ1 in human asthma. The table provided is a compilation of all the studies published so far regarding the expression of TGFβ1 in different lung compartments of human asthmatics. [file 1465-9921-8-66-S1.doc]

Table 1: Expression of TGFβ1 in human asthma

| **Tissues** | **mRNA or protein** | **Description of the effect**  **Asthmatics vs controls** | Ref. |
| --- | --- | --- | --- |
| Lung resections or necropsies | 1- mRNA  (expression relative to GAPDH)  2- Protein | 1- 108 vs 100% in asthmatics vs controls, respectively (ns).  - No difference was observed between specimens obtained from fatal asthmatics (96%) and patients suffering from mild to moderate forms of the disease (121%).  2- Neither different patterns nor different levels of expression. (P.S. control subjects were heavy smokers)  - Connective tissues of the airway wall, alveolar macrophages, airway smooth muscle and the airway epithelium were + for TGFβ1. | [1] |
| Bronchial biopsies | mRNA | - 52.1 vs 10.5 + cells/mm^2^ in severe asthmatics vs controls, respectively (p < 0.02), but was not elevated in mild asthmatics (1.0 + cell/mm^2^)  - In asthmatics, + cells were eo. | [2] |
|  | Protein* | - Altered compartmentalization: In asthmatics, TGFβ intensity of staining decreased in the epithelium (p < 0.01) and increased in the submucosa in association with an increased inflammatory cell infiltrate. | [3] |
|  | Protein* | - Median of 4 vs 0 + cells/mm of BM in the epithelium and 31.5 vs 0 + cells/mm^2^ of BM in the submucosa in asthmatics vs controls, respectively (p ≤ 0.015). | [4] |
|  | 1- mRNA  2- Protein | - 18.5, 10.8 and 7.8 vs 3.5 + cells/mm of BM in severe, moderate and mild asthmatics vs controls, respectively (p < 0.05 for severe and moderate asthmatics).  - 18.8, 12.3 and 9.2 vs 5.2 + cells/mm of BM in severe, moderate and mild asthmatics vs controls, respectively (p < 0.05 for all groups of asthmatics). | [5] |
|  | Protein | - 18 vs 16% relative intensity in the bronchial mucosa of asthmatics vs controls (ns). | [6] |
|  | Protein† | - Neither different patterns nor different levels of expression between asthmatics and controls.  - Positive staining was observed in subepithelial connective tissues of the airway wall and in the bronchial epithelium. | [7] |
|  | Protein* | - 12.5 vs 6.6 + cells/mm^2^ in the submucosa of asthmatics vs controls (ns, p = 0.06). | [8] |
|  | Protein* | - Median of ~41.4 vs 7.8, 11.1, 15, and 8.1 cell/mm^2^ in the submucosa of eo + severe asthmatics vs controls, mild asthmatics, moderate asthmatics and eo – severe asthmatics, respectively (p = 0.0003).  - TGFβ + cells correlate with eosinophils, neutrophils and macrophages number (p < 0.0001). | [9] |
|  | Protein* | - 108 vs 24 + cells/mm^2^ of airway submucosa in asthmatics vs controls (p = 0.002). | [10] |
|  | Protein* | - ~11 and 6 vs < 1 + cells/mm^2^ of bronchial submucosa, in severe-to-moderate and mild asthmatics vs controls, respectively (p < 0.05).  - Immunoreactivity was mainly localized in inflammatory cells. | [11] |
|  | mRNA | - Median of ~43 vs 5 + eo/mm^2^ before vs after treatment with mepolizumab in mild atopic asthmatics, respectively (p = 0.04). - Median of ~20 vs 39 + eo/mm^2^ before vs after placebo treatment, respectively.  - There was no control group for comparison. | [12] |
|  | Protein | - 13.5 vs 3.2% of tissue area in ASM layer in persistent asthmatics vs controls (p = 0.02), but was not elevated in intermittent asthmatics (2.7%) and neither altered in the epithelial and the submucosal tissues. | [13] |
|  | Protein | - Higher intensity of staining in the submucosa, but not in the epithelium, of asthmatics vs controls (p < 0.05).  - TGFβ1 was mainly localized in association with connective tissue in all groups. | [14] |
|  | Protein | - (1.0-2.4) vs (0.2-1.6)% of total epithelial area (interquartile 25-75% range) in asthmatics vs normal subjects, respectively (ns). | [15] |
|  | 1- mRNA  2- Protein* | 1- No difference between mild and severe (with or without persistent eosinophilia) asthmatics vs controls.  2- The majority of subjects in every groups expressed undetectable or very low number of + cells in the submucosa. | [16] |
|  | Protein | - 15.1 vs 14.0% of + cells in the bronchial mucosa of mild atopic asthmatics 24 h after saline vs allergen challenges (ns).  - TGFβ1 was mainly expressed at the apical pole of the epithelium following both the saline and the allergen challenges, but a low percentage (~7%) of eo and neutrophils were equally immunostained.  - There was no comparison with non-asthmatic individuals. | [17] |
|  | 1- mRNA  (Isolation of the ASM tissue by laser capture microdissection before real-time RT-PCR assessment)  2- Protein | 1- Increased expression in asthmatic vs normal individuals (p = 0.029).  2- Mean relative immunostaining intensity in ASM tissue ~doubles in asthmatic vs normal individuals (p = 002). | [18] |
| BALF | Protein | - 8.0 vs 5.5 pg/ml in asthmatics vs controls, respectively (p = 0.027).  - Increased expression following SAC:  31.3 vs 25.0 at 10 min (p = 0.78) and 46.0 vs 21.5 pg/ml at 24 h (p = 0.017) post-allergen- vs saline-challenged sites, respectively. | [19] |
|  | Protein | - Active TGFβ1: median of ~350 and 30 vs 60 pg/ml in status asthmaticus and stable asthmatic patients vs controls, respectively (p < 0.05).  - Latent TGFβ1: median of ~900 and 75 vs 100 pg/ml in status asthmaticus and stable asthmatic patients vs controls, respectively (p < 0.05). | [20] |
|  | Protein | - Median of ~5 vs 4 pg/ml before vs after treatment with mepolizumab in mild atopic asthmatics, respectively (p = 0.05). - Median of ~4 vs 4 pg/ml before vs after placebo treatment, respectively.  - There was no control group for comparison. | [12] |
|  | Protein | - Identical at baseline (~50 pg/ml)  - Significantly increased after SAC in asthmatics only (~170 pg/ml) (p < 0.05). | [21] |
| Sputum | Protein | - Sputum was collected from asthmatics during moderate or severe attacks.  - Active form was ND, but inactive form was 21.7 ng/ml.  - There was no control group for comparison. | [22] |
|  | Protein | - 23 out of 26 asthmatics demonstrated TGFβ1+ cells vs 0 out of 8 in normal volunteers. | [23] |
| Plasma | Protein | - 2.5 vs 1.5 ng/ml in nonatopic asthmatics vs controls, respectively (p = 0.002), but was not different in atopic asthmatics (1.4 ng/ml). | [24] |
| Serum | Protein | - 11.7, 14.5 and 36.8, vs 5.5 ng/ml in severe asthmatics with inhaled and systemic GCs, moderate asthmatics with inhaled GCs and moderate asthmatics without any drug treatment vs healthy subjects, respectively (p ≤ 0.001 for all groups of asthmatics vs controls). | [25] |
| Exhaled breath condensate | Protein* | - 1.69-fold increase in asthmatics compared to healthy subjects (p < 0.01). | [26] |
| Alveolar macrophages | mRNA | - Increased in mild atopic asthmatics vs controls (p < 0.005). | [27] |
|  | Protein | - Median of 435 vs 210 ng/million cells in asthmatics vs controls, respectively (p = 0.005). | [28] |
| Airway epithelial cells | Protein  (*ex vivo*) | - ~4 vs 1.5 pg/10^4^ cells in asthmatics vs controls (p = 0.032). | [29] |
| CD4^+^ T cells | mRNA | Decreased expression in moderate asthmatics without any drug treatment compared to each of the 3 other groups (p ≤ 0.01). | [25] |
| Peripheral blood neutrophils | 1- Protein  (spontaneous release *ex vivo*)  2- mRNA | 1- 115 vs 46 pg/10^6^ cells in asthmatics vs controls, respectively (p = 0.007).  2- Confirmed the expression of TGFβ1 mRNA in neutrophils, but there was no apperance of altered expression levels in asthmatic vs non-asthmatic individuals. | [10] |

*Antibody used did not discriminate between TGFβ1, 2 or 3 or is not specified.

†The question whether the antibody used exhibits cross-reactivity with TGFβ2 or TGFβ3 has not been addressed.

Unless otherwise indicated, amounts of TGFβ1 represent the mean values.

*Abbreviation*: +, positive; BALF, bronchoalveolar lavage fluid; BM, basement membrane; eo, eosinophils; GCs, glucocorticoids; ND, not detected; ns, not statistically significant; SAC, segmental allergen challenge.

# References

1 - Aubert JD, Dalal BI, Bai TR, Roberts CR, Hayashi S, Hogg JC: Transforming growth factor beta 1 gene expression in human airways. Thorax 1994; 49: 225-232.

2 - Ohno I, Nitta Y, Yamauchi K, Hoshi H, Honma M, Woolley K, O'Byrne P, Tamura G, Jordana M, Shirato K: Transforming growth factor beta 1 (TGF beta 1) gene expression by eosinophils in asthmatic airway inflammation. Am J Respir Cell Mol Biol 1996; 15: 404-409.

3 - Magnan A, Retornaz F, Tsicopoulos A, Brisse J, Van Pee D, Gosset P, Chamlian A, Tonnel AB, Vervloet D: Altered compartmentalization of transforming growth factor-beta in asthmatic airways. Clin Exp Allergy 1997; 27: 389-395.

4 - Vignola AM, Chanez P, Chiappara G, Merendino A, Pace E, Rizzo A, la Rocca AM, Bellia V, Bonsignore G, Bousquet J: Transforming growth factor-beta expression in mucosal biopsies in asthma and chronic bronchitis. Am J Respir Crit Care Med 1997; 156: 591-599.

5 - Minshall EM, Leung DY, Martin RJ, Song YL, Cameron L, Ernst P, Hamid Q: Eosinophil-associated TGF-beta1 mRNA expression and airways fibrosis in bronchial asthma. Am J Respir Cell Mol Biol 1997; 17: 326-333.

6 - Hoshino M, Nakamura Y, Sim JJ: Expression of growth factors and remodelling of the airway wall in bronchial asthma. Thorax 1998; 53: 21-27.

7 - Redington AE, Roche WR, Holgate ST, Howarth PH: Co-localization of immunoreactive transforming growth factor-beta 1 and decorin in bronchial biopsies from asthmatic and normal subjects. J Pathol 1998; 186: 410-415.

8 - Chu HW, Halliday JL, Martin RJ, Leung DY, Szefler SJ, Wenzel SE: Collagen deposition in large airways may not differentiate severe asthma from milder forms of the disease. Am J Respir Crit Care Med 1998; 158: 1936-1944.

9 - Wenzel SE, Schwartz LB, Langmack EL, Halliday JL, Trudeau JB, Gibbs RL, Chu HW: Evidence that severe asthma can be divided pathologically into two inflammatory subtypes with distinct physiologic and clinical characteristics. Am J Respir Crit Care Med 1999; 160: 1001-1008.

10 - Chu HW, Trudeau JB, Balzar S, Wenzel SE: Peripheral blood and airway tissue expression of transforming growth factor beta by neutrophils in asthmatic subjects and normal control subjects. J Allergy Clin Immunol 2000; 106: 1115-1123.

11 - Chakir J, Shannon J, Molet S, Fukakusa M, Elias J, Laviolette M, Boulet LP, Hamid Q: Airway remodeling-associated mediators in moderate to severe asthma: effect of steroids on TGF-beta, IL-11, IL-17, and type I and type III collagen expression. J Allergy Clin Immunol 2003; 111: 1293-1298.

12 - Flood-Page P, Menzies-Gow A, Phipps S, Ying S, Wangoo A, Ludwig MS, Barnes N, Robinson D, Kay AB: Anti-IL-5 treatment reduces deposition of ECM proteins in the bronchial subepithelial basement membrane of mild atopic asthmatics. J Clin Invest 2003; 112: 1029-1036.

13 - Berger P, Girodet PO, Begueret H, Ousova O, Perng DW, Marthan R, Walls AF, Tunon de Lara JM: Tryptase-stimulated human airway smooth muscle cells induce cytokine synthesis and mast cell chemotaxis. Faseb J 2003; 17: 2139-2141.

14 - Kokturk N, Tatlicioglu T, Memis L, Akyurek N, Akyol G: Expression of transforming growth factor beta1 in bronchial biopsies in asthma and COPD. J Asthma 2003; 40: 887-893.

15 - Chu HW, Balzar S, Seedorf GJ, Westcott JY, Trudeau JB, Silkoff P, Wenzel SE: Transforming growth factor-beta2 induces bronchial epithelial mucin expression in asthma. Am J Pathol 2004; 165: 1097-1106.

16 - Balzar S, Chu HW, Silkoff P, Cundall M, Trudeau JB, Strand M, Wenzel S: Increased TGF-beta2 in severe asthma with eosinophilia. J Allergy Clin Immunol 2005; 115: 110-117.

17 - Torrego A, Hew M, Oates T, Sukkar M, Fan Chung K: Expression and activation of TGF-beta isoforms in acute allergen-induced remodelling in asthma. Thorax 2007; 62: 307-313.

18 - Xie S, Sukkar MB, Issa R, Khorasani NM, Chung KF: Mechanisms of induction of airway smooth muscle hyperplasia by transforming growth factor-{beta}. Am J Physiol Lung Cell Mol Physiol 2007;

19 - Redington AE, Madden J, Frew AJ, Djukanovic R, Roche WR, Holgate ST, Howarth PH: Transforming growth factor-beta 1 in asthma. Measurement in bronchoalveolar lavage fluid. Am J Respir Crit Care Med 1997; 156: 642-647.

20 - Tillie-Leblond I, Pugin J, Marquette CH, Lamblin C, Saulnier F, Brichet A, Wallaert B, Tonnel AB, Gosset P: Balance between proinflammatory cytokines and their inhibitors in bronchial lavage from patients with status asthmaticus. Am J Respir Crit Care Med 1999; 159: 487-494.

21 - Batra V, Musani AI, Hastie AT, Khurana S, Carpenter KA, Zangrilli JG, Peters SP: Bronchoalveolar lavage fluid concentrations of transforming growth factor (TGF)-beta1, TGF-beta2, interleukin (IL)-4 and IL-13 after segmental allergen challenge and their effects on alpha-smooth muscle actin and collagen III synthesis by primary human lung fibroblasts. Clin Exp Allergy 2004; 34: 437-444.

22 - Adachi T, Motojima S, Hirata A, Fukuda T, Kihara N, Makino S: Detection of transforming growth factor-beta in sputum from patients with bronchial asthma by eosinophil survival assay and enzyme-linked immunosorbent assay. Clin Exp Allergy 1996; 26: 557-562.

23 - Nomura A, Uchida Y, Sakamoto T, Ishii Y, Masuyama K, Morishima Y, Hirano K, Sekizawa K: Increases in collagen type I synthesis in asthma: the role of eosinophils and transforming growth factor-beta. Clin Exp Allergy 2002; 32: 860-865.

24 - Joseph J, Benedict S, Badrinath P, Wassef S, Joseph M, Abdulkhalik S, Nicholls MG: Elevation of plasma transforming growth factor beta1 levels in stable nonatopic asthma. Ann Allergy Asthma Immunol 2003; 91: 472-476.

25 - Karagiannidis C, Hense G, Martin C, Epstein M, Ruckert B, Mantel PY, Menz G, Uhlig S, Blaser K, Schmidt-Weber CB: Activin A is an acute allergen-responsive cytokine and provides a link to TGF-beta-mediated airway remodeling in asthma. J Allergy Clin Immunol 2006; 117: 111-118.

26 - Matsunaga K, Yanagisawa S, Ichikawa T, Ueshima K, Akamatsu K, Hirano T, Nakanishi M, Yamagata T, Minakata Y, Ichinose M: Airway cytokine expression measured by means of protein array in exhaled breath condensate: correlation with physiologic properties in asthmatic patients. J Allergy Clin Immunol 2006; 118: 84-90.

27 - Prieto J, Lensmar C, Roquet A, van der Ploeg I, Gigliotti D, Eklund A, Grunewald J: Increased interleukin-13 mRNA expression in bronchoalveolar lavage cells of atopic patients with mild asthma after repeated low-dose allergen provocations. Respir Med 2000; 94: 806-814.

28 - Vignola AM, Chanez P, Chiappara G, Merendino A, Zinnanti E, Bousquet J, Bellia V, Bonsignore G: Release of transforming growth factor-beta (TGF-beta) and fibronectin by alveolar macrophages in airway diseases. Clin Exp Immunol 1996; 106: 114-119.

29 - Hastie AT, Kraft WK, Nyce KB, Zangrilli JG, Musani AI, Fish JE, Peters SP: Asthmatic epithelial cell proliferation and stimulation of collagen production: human asthmatic epithelial cells stimulate collagen type III production by human lung myofibroblasts after segmental allergen challenge. Am J Respir Crit Care Med 2002; 165: 266-272.
