# Supplementary material for: Controversy surrounding the increased expression of TGFβ1 in asthma
Source: Respir Res. 2007 Sep 24;8(1):66. doi: 10.1186/1465-9921-8-66 (PMC2078588; doi:10.1186/1465-9921-8-66)
Supplement: Additional file 2 — Increased expression of TGFβ1 in animal models of asthma. The table provided is a compilation of published studies regarding the expression of TGFβ1 in different lung compartments of animal models of asthma. [file 1465-9921-8-66-S2.doc]

Table 2: Increased expression of TGFβ1 in animal models of asthma (listed in chronologic order).

| **Species** | **Models** | **Tissues** | **mRNA or Protein** | **Extent of the increase** | Ref. |
| --- | --- | --- | --- | --- | --- |
| Female BALB/c mice | OVA sensitization and challenge | Whole-lung lavage | Active protein | - Increased in function of allergen concentration. From ~10, 60, 145 and 190 pg/ml in saline, 0.01, 0.1 and 1% OVA, respectively (p < 0.01). | [1] |
| C57BL/6 mice | IL-13 transgenic | 1- Lung homogenates  2- Whole-lung lavage  3- Lung sections | 1- mRNA  2a- Protein  2b- Active protein  3- mRNA and Protein | 1- Increased expression in transgenic mice vs littermate controls.  2a- ~294 vs 132, 1794 vs 59 and 2610 vs 88 pg/ml in transgenic mice vs littermate controls at 1, 2 and 3 mo of age, respectively (p < 0.01).  2b- ~2.4 to 4.3-fold increases in luciferase activity when CCI-64 cells stably transfected with a luciferase reporter gene driven by the PAI-1 promoter were stimulated with BALF of transgenic compared to littermate control mice (p < 0.001).  3- Expression was restricted to the airway epithelium and to some AM in littermate controls, but increased in AM and was appreciated in airway epithelium, type II pneumocytes and occasionally in eosinophils of transgenic mice. | [2] |
| Male C3H/HeBFeJ mice | Endotoxin-induced asthma | Lung sections  1- Subepithelium area  2- Epithelium | Protein  (Staining intensity was graded from 0 to 3) | 1- 2.64 vs 0 relative intensity in LPS- vs air-exposed animals after 4 wk of LPS exposure (p < 0.005), but this difference did not persist after a 4 wk recovery period.  2- 2.59 vs 0.83 relative intensity in LPS- vs air-exposed animals after 4 wk of recovery of the 4 wk LPS exposure (p < 0.005), but no difference (0.83 vs 1.14 in air- vs LPS-exposed animals) after the 4 wk of exposure. | [3] |
| Female BALB/c, BALB/cJ, and BALB/cJ congenic IL-4 KO mice | OVA sensitization and challenge | Whole-lung lavage | Active protein | - 17.1 and 265.3 pg/ml in sensitized only and sensitized/challenged BALB/c, respectively (p < 0.01).  - 27.5, 32.5 and 333.8 pg/ml in control, sensitized only and sensitized/challenged BALB/cJ, respectively (p < 0.01).  - 27.9, 29.5 and 81.6 pg/ml in control, sensitized only and sensitized/challenged IL-4 KO, respectively (p < 0.01). | [4] |
| Male BP2 mice | OVA sensitization and challenge | - BALF and whole lung | Protein* | - Increased expression in both of these lung compartments 48 h after the last challenge of a 8 mo protocol of bronchoprovocation, but not after 48 h of a single challenge, compared to control mice. | [5] |
| Female WBB6F_1_, W/W^V^ and S1/S1^d^ mice | OVA sensitization and challenge | Whole-lung lavage | Active protein | - 44.9, 41.8 and 583.6 pg/ml in control, sensitized only and sensitized/challenged WBB6F_1_ mice, respectively (p < 0.001).  - 38.4, 45.3 and 555.8 pg/ml in control, sensitized only and sensitized/challenged W/W^V^, respectively (p < 0.001).  - 102.4 vs 464.6 pg/ml in sensitized only vs sensitized/challenged Sl/Sl^d^, respectively (p < 0.05). | [6] |
| Male C3HeB/FeJ (LPS-sensible) and C3H/HeJ (LPS-insensible)  mice | Endotoxin-induced asthma | Whole-lung lavage | 1- Protein  2- Active protein | 1- ~865 vs 58 pg/ml in C3HeB/FeJ vs C3H/HeJ after 5 d of LPS exposure (p < 0.05), but was not different after 4 h of exposure or 96 h after an 8 wk of exposure.  2- ~111 vs 19 pg/ml in C3HeB/FeJ vs C3H/HeJ after 5 d of LPS exposure (p < 0.05), and ~22 vs 0 pg/ml in C3HeB/FeJ vs C3H/HeJ after 4 h of LPS exposure (p < 0.05), but not different 96 h after a 8 wk of LPS exposure. | [7] |
| Female C57BL/6 and congenic IP KO mice | OVA sensitization and challenge | Whole-lung lavage | Active protein | - 47.5, 57.9 and 178.0 pg/ml in control, sensitized only and sensitized/challenged C57BL/6, respectively (p < 0.01).  - 39.1, 44.3 and 342.5 pg/ml in control, sensitized only and sensitized/challenged IP KO, respectively (p < 0.01). | [8] |
| C57BL/6 and congenic IL-5 KO  mice | OVA sensitization and challenge | 1- Lung homogenates  2- Lung sections  (number of peribronchial cells + for TGFβ per bronchiole of 150-200 µm of internal diameter) | 1- Protein  2- Protein* | 1- 3740 vs ~1855 pg/ml in challenged vs unchallenged sensitized WT mice, respectively (p < 0.01).  - 2170 vs ~1455 pg/ml in challenged vs unchallenged sensitized IL-5^-/-^ mice (p < 0.05 when compared challenged mice with WT and IL-5^-/-^ genotypes).  2- 64.5 vs ~5 TGFβ + cells/bronchus in challenged vs unchallenged sensitized WT mice, respectively (p < 0.001).  - 22.6 vs ~1 TGFβ + cells/bronchus in challenged vs unchallenged sensitized IL-5^-/-^ mice (p < 0.001 when compared challenged mice with WT and IL-5^-/-^ genotypes).  - Peribronchial cells + for TGFβ were mainly eo (63%) and macrophages (35%), but increased expression of TGFβ was also observed in the epithelium of both WT and IL-5^-/-^ challenged mice. | [9] |
| Female BALB/c mice | OVA sensitization and challenge | 1- Lung sections  2- Lung homogenates | 1- Protein  2- Active protein | 1- Altered compartmentalization of TGFβ1 immunoreactivity, from airway epithelium to submucosal compartment.  2- ~66, 180, 160 and 66 vs 50 pg/ml in 25, 35, 55 days challenged and 80 days challenged, 1 month recovered vs control mice, respectively (p < 0.05 for 35 and 55 days challenged vs control mice). | [10] |
| Female BALB/c, congenic IL-13^-/-^ and CD4^+^-depleted  mice | OVA sensitization and acutely challenge  or chronically challenge | 1- Lung sections  2- Whole-lung lavage | 1a- Protein (Ab used was specific for the active form, but the latent form was revealed following proteinase K treatment)  (staining intensity was graded from 0 to 3)  1b- Active protein  2-Active protein | 1a- Intense staining in airway epithelium of naïve mice  - Median values of 3 vs 2 in the epithelium of chronically challenged (6 wk), sensitized WT vs similarly treated IL-13^-/-^ mice (p < 0.01).  - Median values of 3 vs 3 in the epithelium of chronically challenged (6 wk), sensitized WT vs similarly treated CD4^+^-depleted mice (ns).  1b- Median values of 2, 1.5 and 0 in the epithelium at 3, 6 and 24 h following the last exposure, respectively, in chronically challenged, sensitized WT mice compared to 0, 0 and 0.5 in acutely challenged, non-sensitized and to 1, 1.5 and 0.5 in acutely challenged, sensitized WT mice at the same time points.  - Median values of 2, 2 and 2 in the subepithelial zone at 3, 6 and 24 h following the last exposure, respectively, in chronically challenged, sensitized WT mice compared to 0, 0 and 0 in acutely challenged, non-sensitized and 2, 3 and 2 in acutely challenged, sensitized WT mice at the same time points.  - Median values of 3, 2, 2 and 1 vs 0 in the subepithelial zone of sensitized WT mice, challenged for 8, 6, 4 and 2 wk vs naïve mice, respectively (p < 0.05 for 8 and 6 wk compared to naïve).  - Median values of 2 vs 1 in the subepithelial zone of chronically challenged (6 wk), sensitized WT vs similarly treated IL-13^-/-^ mice (p < 0.05).  - Median values of 2 vs 0 in the epithelium of chronically challenged (6 wk), sensitized WT vs similarly treated IL-13^-/-^ mice 3 h after the last exposure (p < 0.01).  - Median values of 2 vs 0.5 in the subepithelial zone of chronically challenged (6 wk), sensitized WT vs similarly treated CD4^+^-depleted mice (p < 0.01).  2- 11.8, 6.5, 4.3 and 8.0 vs 5.1, ng/ml in non-sensitized acute, sensitized acute, non-sensitized chronic and sensitized chronic at 6 h following the last challenge vs naïve, respectively (ns).  - 80% of TGFβ1 in the BALF was in an active form. | [11] |
| Female BALB/c mice | OVA sensitization and challenge | 1- Whole-lung lavage fluid  2- Lung homogenates | Protein | 1- 300 vs 135, 356 vs 156 and 369 vs 146 pg/ml in sensitized mice challenged vs unchallenged for 1, 3 and 6 mo, respectively (p = 0.03).  - Reduction to 241 and 269 pg/ml with ISS treatment in sensitized mice challenged for 3 mo and 6 mo, respectively, (p = 0.05), but no reduction in the 1 mo challenged group.  2- 1946 vs 664 pg/mg of lung protein in challenged (3 mo) vs unchallenged sensitized mice, respectively (p = 0.02).  - Reduction to 939 pg/mg of lung protein in challenged sensitized mice treated with ISS (p = 0.05) | [12] |
| Male C57BL/6 and congenic LBP KO mice | Endotoxin-induced asthma | Whole-lung lavage fluid | 1- Protein  2- Active protein | 1- 266.1, 173.8 and 43.2 vs nd pg/ml in 4 wk exposed, 3 d recovered, 5 d and 4 h exposed vs control mice, respectively.  - 115.7, 87.7 and 34.2 vs nd pg/ml in 4 wk exposed, 3 d recovered, 5 d and 4 h exposed vs control LBP^-/-^ mice, respectively.  2- 4.3, 58.3 and 6.5 vs nd pg/ml in 4 wk exposed, 3 d recovered, 5 d and 4 h exposed vs control mice, respectively.  - nd, 23.5 and 5.4 vs nd pg/ml in 4 wk exposed, 3 d recovered, 5 d and 4 h exposed vs control LBP^-/-^ mice, respectively. | [13] |
| Female BALB/c mice | OVA sensitization and challenge | 1- Whole lung  2- Microdissected: a) Bronchial wall  b) Epithelium  c) ASM | mRNA | 1- No difference between sensitized mice challenged with OVA or saline at either 2 or 8 wk following the last exposure.  2- Increased at 2 wk, but not at 8 wk, following the last exposure in the bronchial wall (p < 0.05).  - When the epithelium and the ASM cells of the bronchial wall were analysed separately, the increase observed at 2 wk after the last exposure was restricted to the epithelium (p < 0.05). | [14] |
| Male BALB/c mice | OVA sensitization and challenge | Whole-lung lavage | Active Protein* | ~300 vs 30 pg/ml in sensitized and challenged vs shammed animals, (ns). | [15] |
| BALB/c mice | OVA sensitization and challenge | Lung homogenates | mRNA | - Weak, but seemingly significant decrease at 2, but not 12 h, following two or four 1-h antigen challenge in mice recovered from a first period of challenge 228 days ago (p ≤ 0.01). | [16] |
| BALB/c  T-cell GATA-3 or T-bet transgenic mice | OVA sensitization and challenge | Lung homogenates | Protein | - ~6000 vs 7250 and 5250 pg/rat lung in WT vs GATA and T-bet transgenic naïve mice, respectively, compared to 5750 vs 8375 and 4425 in the same groups 1 d following the last challenge on sensitized mice (p < 0.05 for both transgenic mice vs WT after sensitization/challenge). | [17] |
| Brown-Norway rats | OVA sensitization and challenge | - Lung sections (midlevel segmental bronchi) | Protein* | - Staining intensity scores in airway epithelium and ASM tissue were significantly increased compared to isotype control staining in sensitized/challenged (4.71), but not in control rats (2.00).  - Addition of pirfenidone in sensitized/challenged animal’s diet may slightly decreased staining intensity (4.5). | [18] |
| Female BALB/c mice | Occupational asthma (TDI-induced) | 1- Lung homogenates  2- Whole-lung lavage | Protein | 1- Increased expression 48 h after the last TDI inhalation.  - PPARγ agonists rosiglitazone and pioglitazone, as well as transferred of adenovirus gene vector expressing PPARγ2 cDNA or BAY 11-7085 partially prevent TDI-induced TGFβ1.  2- nd vs ~120 pg/ml 48 h after the last TDI inhalation in control vs experimental group, respectively (p < 0.05).  - PPARγ agonists rosiglitazone and pioglitazone, as well as transferred of adenovirus gene vector expressing PPARγ2 cDNA partially prevent TDI-induced TGFβ1 (p < 0.05). | [19] |
| Female BALB/c mice | OVA sensitization and challenge | 1- Whole-lung lavage  2- Lung sections | Protein | 1- 236 vs 789 and 543 pg/ml in control vs sensitized/challenged mice treated or not with fluticasone, respectively (p < 0.05).  2- Increased positive cells in the peribronchial region of sensitized/challenged vs control mice, which was reduced by fluticasone treatment. | [20] |
| Female BALB/c mice | OVA sensitization and challenge | Whole-lung lavage | Protein | - ~650 vs 450 pg/ml in OVA-challenged vs saline-challenged sensitized mice, respectively.  - Continued to increase steadily in a chronic (38 additional days) challenge protocol, up to ~900 pg/ml.  - Correlated with eo counts in the lavage (R^2^ = 0.89).  - The increase was completely reversed with a bispecific antibody directed against CD300a that targeted CCR3+ cells (LC1) (p < 0.01). | [21] |
| Female  C57BL/6 | OVA sensitization and challenge | 1- Whole-lung lavage (7 days after the last challenge)  2- Lung homogenates (7 days after the last challenge) | Protein | 1- ~125 vs nd pg/ml in OVA-challenged vs saline-challenged sensitized mice, respectively (p < 0.05).  - Reduced to ~60, 65 and 70 vs 126 or 128 pg/ml in Montelukast-, Pranlukast- and anti-IL-11 Ab- vs vehicle- or control Ab-treated animals, respectively (p < 0.05).  2- Increased in OVA-challenged vs saline-challenged sensitized mice (p < 0.05).  - Reduced in Montelukast-, Pranlukast- and anti-IL-11 Ab- vs vehicle- or control Ab-treated animals (p < 0.05). | [22] |
| BALB/c mice | OVA sensitization and challenge with or without ETS exposure | Lung sections | Protein | - 12.1 vs 0.3 + cells/bronchus in OVA vs non-OVA sensitized mice, respectively (p = 0.01).  - ETS alone did not affect the number of peribronchial + cells.  - ETS amplified the effect of OVA challenge in sensitized mice (~39 + cells/bronchus; p = 005). | [23] |

*Antibody used did not discriminate between TGFβ1, 2 or 3 or is not specified.

Unless otherwise indicated, amounts of TGFβ1 represent the mean values.

*Abbreviation*: Ab, antibody; AM, alveolar macrophages; ASM, airway smooth muscle; d, day; eo, eosinophils; ETS, environmental tobacco smoke; h, hour; IP, prostaglandin (PG)I_2_ receptor; ISS, immunostimulatory sequences of DNA; KO, knockout; LBP, LPS binding protein; nd, none detected; ns, not statistically significant; OVA, ovalbumin; PAI-1, plasminogen activator inhibitor-1; PPAR, peroxisome proliferator-activated receptor; TDI, toluene diisocyanate; wk, week; WT, wild type.

### References

1 - Tanaka H, Masuda T, Tokuoka S, Komai M, Nagao K, Takahashi Y, Nagai H: The effect of allergen-induced airway inflammation on airway remodeling in a murine model of allergic asthma. Inflamm Res 2001; 50: 616-624.

2 - Lee CG, Homer RJ, Zhu Z, Lanone S, Wang X, Koteliansky V, Shipley JM, Gotwals P, Noble P, Chen Q, Senior RM, Elias JA: Interleukin-13 induces tissue fibrosis by selectively stimulating and activating transforming growth factor beta(1). J Exp Med 2001; 194: 809-821.

3 - Savov JD, Gavett SH, Brass DM, Costa DL, Schwartz DA: Neutrophils play a critical role in development of LPS-induced airway disease. Am J Physiol Lung Cell Mol Physiol 2002; 283: L952-962.

4 - Komai M, Tanaka H, Masuda T, Nagao K, Ishizaki M, Sawada M, Nagai H: Role of Th2 responses in the development of allergen-induced airway remodelling in a murine model of allergic asthma. Br J Pharmacol 2003; 138: 912-920.

5 - Corbel M, Caulet-Maugendre S, Germain N, Lagente V, Boichot E: Enhancement of gelatinase activity during development of subepithelial fibrosis in a murine model of asthma. Clin Exp Allergy 2003; 33: 696-704.

6 - Masuda T, Tanaka H, Komai M, Nagao K, Ishizaki M, Kajiwara D, Nagai H: Mast cells play a partial role in allergen-induced subepithelial fibrosis in a murine model of allergic asthma. Clin Exp Allergy 2003; 33: 705-713.

7 - Brass DM, Savov JD, Gavett SH, Haykal-Coates N, Schwartz DA: Subchronic endotoxin inhalation causes persistent airway disease. Am J Physiol Lung Cell Mol Physiol 2003; 285: L755-761.

8 - Nagao K, Tanaka H, Komai M, Masuda T, Narumiya S, Nagai H: Role of prostaglandin I2 in airway remodeling induced by repeated allergen challenge in mice. Am J Respir Cell Mol Biol 2003; 29: 314-320.

9 - Cho JY, Miller M, Baek KJ, Han JW, Nayar J, Lee SY, McElwain K, McElwain S, Friedman S, Broide DH: Inhibition of airway remodeling in IL-5-deficient mice. J Clin Invest 2004; 113: 551-560.

10 - McMillan SJ, Lloyd CM: Prolonged allergen challenge in mice leads to persistent airway remodelling. Clin Exp Allergy 2004; 34: 497-507.

11 - Kumar RK, Herbert C, Foster PS: Expression of growth factors by airway epithelial cells in a model of chronic asthma: regulation and relationship to subepithelial fibrosis. Clin Exp Allergy 2004; 34: 567-575.

12 - Cho JY, Miller M, Baek KJ, Han JW, Nayar J, Rodriguez M, Lee SY, McElwain K, McElwain S, Raz E, Broide DH: Immunostimulatory DNA inhibits transforming growth factor-beta expression and airway remodeling. Am J Respir Cell Mol Biol 2004; 30: 651-661.

13 - Brass DM, Savov JD, Whitehead GS, Maxwell AB, Schwartz DA: LPS binding protein is important in the airway response to inhaled endotoxin. J Allergy Clin Immunol 2004; 114: 586-592.

14 - Kelly MM, Leigh R, Bonniaud P, Ellis R, Wattie J, Smith MJ, Martin G, Panju M, Inman MD, Gauldie J: Epithelial expression of profibrotic mediators in a model of allergen-induced airway remodeling. Am J Respir Cell Mol Biol 2005; 32: 99-107.

15 - Peng T, Hao L, Madri JA, Su X, Elias JA, Stahl GL, Squinto S, Wang Y: Role of C5 in the development of airway inflammation, airway hyperresponsiveness, and ongoing airway response. J Clin Invest 2005; 115: 1590-1600.

16 - Karagiannidis C, Hense G, Martin C, Epstein M, Ruckert B, Mantel PY, Menz G, Uhlig S, Blaser K, Schmidt-Weber CB: Activin A is an acute allergen-responsive cytokine and provides a link to TGF-beta-mediated airway remodeling in asthma. J Allergy Clin Immunol 2006; 117: 111-118.

17 - Kiwamoto T, Ishii Y, Morishima Y, Yoh K, Maeda A, Ishizaki K, Iizuka T, Hegab AE, Matsuno Y, Homma S, Nomura A, Sakamoto T, Takahashi S, Sekizawa K: Transcription factors T-bet and GATA-3 regulate development of airway remodeling. Am J Respir Crit Care Med 2006; 174: 142-151.

18 - Mansoor JK, Decile KC, Giri SN, Pinkerton KE, Walby WF, Bratt JM, Grewal H, Margolin SB, Schelegle ES: Influence of pirfenidone on airway hyperresponsiveness and inflammation in a Brown-Norway rat model of asthma. Pulm Pharmacol Ther 2006; In press.

19 - Lee KS, Park SJ, Kim SR, Min KH, Jin SM, Lee HK, Lee YC: Modulation of airway remodeling and airway inflammation by peroxisome proliferator-activated receptor gamma in a murine model of toluene diisocyanate-induced asthma. J Immunol 2006; 177: 5248-5257.

20 - Lee SY, Kim JS, Lee JM, Kwon SS, Kim KH, Moon HS, Song JS, Park SH, Kim YK: Inhaled corticosteroid prevents the thickening of airway smooth muscle in murine model of chronic asthma. Pulm Pharmacol Ther 2006; In press.

21 - Munitz A, Bachelet I, Levi-Schaffer F: Reversal of airway inflammation and remodeling in asthma by a bispecific antibody fragment linking CCR3 to CD300a. J Allergy Clin Immunol 2006; 118: 1082-1089.

22 - Lee KS, Kim SR, Park HS, Park SJ, Min KH, Lee KY, Jin SM, Lee YC: Cysteinyl leukotriene upregulates IL-11 expression in allergic airway disease of mice. J Allergy Clin Immunol 2007; 119: 141-149.

23 - Min MG, Song DJ, Miller M, Cho JY, McElwain S, Ferguson P, Broide DH: Coexposure to environmental tobacco smoke increases levels of allergen-induced airway remodeling in mice. J Immunol 2007; 178: 5321-5328.
